# Prediction of relevant biomedical documents: a human microbiome case study

**DOI:** 10.1186/s13040-015-0061-5

**Published:** 2015-09-10

**Authors:** Paul Thompson, Juliette C. Madan, Jason H. Moore

**Affiliations:** 1 Program in Linguistics, Dartmouth College, Hanover, NH 03755 USA; 2 Department of Pediatrics, Division of Neonatology, Dartmouth-Hitchcock Medical Center, One Medical Center Drive, Lebanon, NH 03756 USA; 3 Institute for Biomedical Informatics, Departments of Genetics and Biostatistics and Epidemiology, Perelman School of Medicine, University of Pennsylvania, 3535 Market Street, Philadelphia, PA 19104 USA

## Abstract

**Background:**

Retrieving relevant biomedical literature has become increasingly difficult due to the large volume and rapid growth of biomedical publication. A query to a biomedical retrieval system often retrieves hundreds of results. Since the searcher will not likely consider all of these documents, ranking the documents is important. Ranking by recency, as PubMed does, takes into account only one factor indicating potential relevance. This study explores the use of the searcher’s relevance feedback judgments to support relevance ranking based on features more general than recency.

**Results:**

It was found that the researcher’s relevance judgments could be used to accurately predict the relevance of additional documents: both using tenfold cross-validation and by training on publications from 2008–2010 and testing on documents from 2011.

**Conclusions:**

This case study has shown the promise for relevance feedback to improve biomedical document retrieval. A researcher’s judgments as to which initially retrieved documents are relevant, or not, can be leveraged to predict additional relevant documents.

## Background

### Manual curation, document ranking, and relevance feedback

When a person judges a document to be relevant to a particular query, or information need, such a judgment is called a relevance judgment. Relevance judgments have been used to evaluate document retrieval systems since the 1950s [1] up until the present [2]. Relevance judgments are used to construct ground truth test collections, so that the retrieval accuracy of retrieval methods can be evaluated. Ground truth test collections for the evaluation of document retrieval systems have sets of queries and documents for which human experts have judged the relevance of document query pairs. [3]. Relevance judgments can also be used to provide relevance feedback [4, 5]. With relevance feedback a first iteration of documents retrieved by a retrieval system is evaluated for relevance, e.g., the searcher makes relevance judgments for each retrieved document. This feedback enables the weights that a retrieval system uses to rank documents to be adjusted. The system then retrieves another set of documents based on these adjusted weights. This process can be iterated multiple times. Laboratory studies have shown that relevance feedback can lead to much more effective document retrieval, as measured by the standard metrics of precision and recall [3]. For further background on document retrieval research see also [6–8].

The 1990s was a time of innovation in the development of large-scale information retrieval systems in the medical, legal, and aerospace domains and on the Web. Until that time academic research on information retrieval had little connection with commercial online information retrieval systems. The commercial systems all used Boolean exact match retrieval, while academic researchers studied vector space or probabilistic retrieval models, which ranked documents according to relevance in response to a user’s query. In 1992, West Publishing Company, the legal publisher, introduced a ranked retrieval search mode for its Westlaw retrieval system. Westlaw’s probabilistic retrieval algorithm is based on Bayesian belief networks [9]. Every point of law in each of West’s caselaw documents is manually identified by an attorney editor and categorized according to the KeyNumber System, a taxonomy of approximately 100,000 categories with up to eight levels of hierarchy [10].

Lexis-Nexis, the other major legal retrieval system, and DIALOG, a widely-used retrieval service, also developed ranked retrieval search modes within a year. These three were the first large-scale commercial retrieval systems providing relevance ranking. Any document retrieval system that displays lists of retrieved documents in response to a query needs to rank documents. Before the 1990s, however, commercial systems did not rank based on relevance, but rather ranked based on some objective feature of the document, e.g., its recency of publication. Biomedical searchers are often interested in finding the most recent papers, but recency is only one factor that might be used to predict relevance. Searchers using each of these three retrieval systems, however, had the option of using the traditional Boolean search mode as well, rather than the new ranked retrieval mode, and the vast majority of users continued to use Boolean search. At the National Aeronautics and Space Administration (NASA) and at the European Institute of Cognitive Sciences and Engineering (EURISCO) research projects during this same period focused on developing machine learning algorithms to model the information needs of individual searchers who searched through technical documentation on a daily basis [11–14].

While informatics researchers in the biomedical domain eventually explored many techniques for information retrieval and extraction, as well as automated curation, retrieval systems, such as PubMed [15], the National Library of Medicine’s document retrieval system, do not offer relevance-ranked retrieval search modes. However, over the past 15 years many new information retrieval tools have been introduced to complement PubMed information retrieval [16]. Furthermore, the National Library of Medicine developed the Unified Medical Language System (UMLS) [17], and many other taxonomies and ontologies were developed by various groups. Despite the focus in the biomedical domain on ontology development and manual curation, it is recognized that the rate at which biomedical literature is being produced is too great for manual curation to keep pace [18].

During this same time the World Wide Web was introduced, and there were soon Web search engines. Early Web search engines used the same ranked retrieval approaches developed in academia and implemented in legal and other search services. These approaches did not work well on the Web and were soon replaced by new algorithms, such as Hubs and Authorities [19], or PageRank [20], that ranked documents based on the link structure of the Web. Using these algorithms a document receives a high ranking if it has all of the user’s search terms (often only one or two terms are used in Web searching) and if it is linked to by many other popular Web sites.

Ontologies and taxonomies, such as UMLS or the KeyNumber system are costly to develop and maintain. Furthermore, a searcher may not view her, or his, research area in the same way as the creators of ontologies or taxonomies. In a rapidly developing field such as the human microbiome, terminology may evolve quickly. The attraction of ranked retrieval algorithms is that they can make use of the full text of documents to rank documents for searchers. The example of early Web search engines shows that having the full text may not be enough. A Web search would typically return, say, 5,000,000 results with a very poor ranking of the documents. A similar point was shown in the 1970s for the legal domain, when full text retrieval systems first became available [21]. Attorneys and paralegals who thought that they were retrieving at least 75 % of all relevant documents for a court case, were at best finding only about 20 % of the relevant documents.

Laboratory experiments in academia have often shown dramatic gains from using relevance feedback [4]. Once ranked retrieval was used commercially, however, relevance feedback was either not used, or used in a very limited way. Even in academic research, there was a trend away from using real relevance judgments, which were seen as too costly to obtain. The Text REtrieval Conference (TREC), is an annual benchmark retrieval challenge task with multiple tracks which has been held every year since 1992 by the National Institute of Standards and Technology [2]. At the TREC conference there has been much experimentation with pseudo-relevance feedback in which the assumption is made that the top *n* ranked documents are relevant and then proceeding as though a user had judged each of these *n* documents to be relevant. From 2008 to 2010 the TREC conference held a relevance feedback track [5]. Despite the strong belief of many information retrieval researchers in the effectiveness of relevance feedback, this track was not long lived.

#### Relevance judgments in the evaluation and operation of document retrieval systems

An issue with most relevance feedback studies, as well as with evaluations of retrieval systems more generally, is that relevance judgments are not provided by real users with real information needs. Rather, as with the evaluations done for the Text REtrieval Conference, or, TREC [2], one or more non-users judge whether the document should be considered relevant or not. Saracevic describes the manner in which relevance judgments have been used in information retrieval evaluation [22].

The objective of relevance judgments in IR tests is to get as close as possible to real-life situations so that test results would have real-life validity. This is very, very difficult to achieve. Thus, simulation methods have been developed. Basically, there are four methods by which relevance judgments have been obtained that are regarded as gold standards:

1. By the user or questioner—person who posed own question made the judgment as well;
2. By a user surrogate(s)—such as a specialist (or by consensus of a group of specialists) who perform judgments on the topic of a given question in their specialty;
3. By an information professional (or by consensus of a group of professionals) who is professionally entrusted or involved with some aspect of the process, who performs judgments on the topic of a given question that is not necessarily in their specialty, but is familiar with what is going on; and
4. By “bystanders” signifying none of the above—for example, by students asked to do a given task of judgment, including possible prescreening.

The first method involves “real users” and the others “laboratory-type users”.

Very few evaluations of document retrieval systems have used relevance judgments as described in method one. For example, the TREC conference primarily uses method two and, to some extent, methods three and four. For previous evaluations using method one, Saracevic mentions only Lancaster’s evaluation of the National Library of Medicine’s Medical Literature Analsyis and Retrieval System (MEDLARS) [23] and Saracevic’s own evaluations of the DIALOG system [24–26]. Organizations have felt that it was too expensive and time consuming to use method one. On the other hand, relevance feedback judgments have be used with operational information retrieval systems. The legal information retrieval system Lexis-Nexis has supported relevance feedback, at least to the extent that a user has been able to make a relevance judgment for a single highly relevant document and the system has used this feedback for another iteration of ranked retrieval. Some early World Wide Web search engines also had similar functionality, i.e., a “more like this” command where a user could provide relevance feedback based on a single relevant document. Spink et al. [27] analyzed the relevance feedback capability of the Excite web search engine. They found that the relevance feedback mechanism was effective in retrieving further relevant documents, but that few searchers used it. More recently two relevance feedback engines have been developed for use with PubMed. RefMed [28] provides a multi-level relevance feedback capability with which searchers can make explicit (i.e., the searcher selects both which documents are relevant and non-relevant) relevance judgments for documents retrieved by a PubMed search. Then a second iteration of documents are retrieved based on this feedback. The searcher can do as many of these iterations as desired to find more relevant documents. MiSearch [29] on the other hand, takes as its starting point the notion documented by Spink et al. [27] and many others that few searchers will be willing to take the time to provide explicit relevance feedback, even if doing so would lead to finding more relevant documents. Instead MiSsearch records implicit relevance feedback [30] by observing the searcher's past behavior when viewing retrieved documents. These observations are used to build a statistical profile of the searcher that is then used to rerank search results from a PubMed query.

### Document retrieval and information needs

The information retrieval problem is to find the right document, or documents, that will help satisfy a searcher’s information need. While there are many types of information need, e.g., to find a known item, more generally, a more complex information need can be regarded as a mental state representing a gap in the searcher’s knowledge. Information found in one or more documents, which may satisfy this information need, is an expression of knowledge represented in the mind of the author of the document. An information retrieval system, such as PubMed, of course, has access to neither the searcher’s information need, nor the knowledge in the mind of the author. Instead, the retrieval system has the searcher’s query and some representation of the document, say an abstract and descriptors, or possibly the full text of the document.

Relevance is one of the fundamental concepts of information retrieval. Many definitions have been proposed [31–33]. Many information retrieval experiments have taken relevance to be a relation between a query and a document, but an arguably more accurate view is that relevance is a relation between an information need and the intellectual content represented in a document [34]. The searcher’s query is a representation of the searcher’s information need. This need may be expressed in multiple queries, but still each of these queries is derived from the searcher’s information need.

In the past most searching, particularly in the biomedical and legal domains, was done by professional searchers. Often these searchers were trained reference librarians who engaged in a reference interview with the researcher who needed the information so that the searcher could express the researcher’s information need correctly, usually as a Boolean logic expression with search terms connected with logical operators such as AND, OR, or NOT [35]. For example, the query “gut AND microbiome” would retrieve all documents that included both the words “gut” and “microbiome”. Furthermore, medical indexers, or attorney editors, who were in both instances highly trained professionals, assigned metadata to documents, such as Medical Subject Headings, which allowed documents to be retrieved based on the indexer’s expert understanding of the document’s intellectual content.

Today manual curation in the medical and legal fields, i.e., the assignment of metadata to documents by domain experts, as described above, continues, though generally a researcher does her or his own searching. Traditional retrieval systems were bibliographic retrieval systems. The record for each document only included metadata. If any of the textual content of the document were included, it was only the abstract. In today’s retrieval systems, the full-text of the document is usually available to search. This is true of Web search engines as well as legal information retrieval systems and medical retrieval systems such as PubMed Central [36]. While Web search engines sometimes allow Boolean search operators, most Web searches do not include such operators. Rather documents are ranked based on some metric involving word frequencies in the documents or on patterns of linkage among websites [19, 20].

### A role for relevance feedback in human microbiome literature searching

The study of the human microbiome is a cross-disciplinary, rapidly evolving field. As such it can be difficult for a clinical researcher to find relevant papers from the literature. Medical literature search can be cumbersome, incomplete, and produce “islands” of references that are not integrated or miss large amounts of relevant data. Arguably, relevance feedback can help alleviate this problem. Once a researcher has identified, through the provision of relevance judgments, a seed collection of relevant, and non-relevant documents for her or his research, these documents can be used as a model of that researcher’s information need, which is potentially a much more powerful representation of the researcher’s information need than the PubMed queries which were used to retrieve the documents for review. These queries retrieve documents that match the specification of the query, but which may, or may not, be relevant. Once a researcher has indicated which documents are in fact relevant, the relevant, and non-relevant documents can be used as positive and negative examples for machine learning algorithms to predict the relevance of additional documents. By reading each relevant and non-relevant document a person with domain knowledge could gain a good understanding of what constituted a relevant document for this researcher.

Our research seeks to establish whether an automated approach could simulate this understanding, i.e., of what distinguishes a relevant from a non-relevant document for this researcher, in order to predict which as yet unseen documents the researcher would judge relevant. The goal of this study is to show that the words, and their within document and overall frequencies within the collection of reviewed documents, taken from documents judged to be relevant and non-relevant to her information need by a human microbiome researcher, can be used to predict the relevance and non-relevance of additional documents to her information need. This technique is called relevance feedback. While we have not implemented relevance feedback in an operational document retrieval system, we have provided a case study of the effectiveness of a relevance feedback component of an operational system.

Our initial hypothesis was that we could use our searcher’s relevance judgments on the approximately three quarters of the documents published during 2008–2010 to predict her judgments for the quarter of the documents published in 2011. Since the field of human microbiome research is evolving very rapidly, we thought that the language of the 2008–2010 documents might not predict which documents from 2011 were relevant, as well as might be the case if the field were not so rapidly evolving. Accordingly, we also did another set of experiments in which we pooled all of the documents and used ten-fold cross-validation.

## Methods

**Fig. 1:**
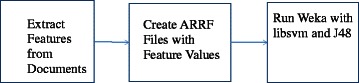
Machine learning steps

Figure 1 shows the steps for our study. First we obtained the full text of the publications found with the PubMed queries. In some cases the full-text of the publication is available through PubMedCenteral. In other cases we were able to obtain the full-text through the publishers through Dartmouth Libraries licenses. Once the full-text of the document was available, we extracted the words from the text. We created features by removing stop words and using an automatic indexing algorithm to produce the counts for the each feature, i.e., the stemmed forms of words. For our machine learning experiments we used the WEKA machine learning toolkit [37]. We used two machine learning algorithms: C4.5 (J48 in WEKA) and Support Vector Machines (SVMs) (libsvm in WEKA). C4.5 is a standard decision tree-based machine learning algorithm which is often used as a baseline against which to compare results obtained with other algorithms [38]. SVMs are a more complex approach, which often gives excellent performance for text categorization [39]. These feature values, the word counts, were then placed in Weka’s Attribute-Relations File Format (ARRF). Then the Weka machine learning algorithms, J48 and libsvm, were run to produce our binary categorization results.

For this study we retrieved 658 documents, using six PubMed queries, for one of the authors, a neonatal human microbiome researcher. She judged each one of these documents as relevant, possibly relevant, or not relevant. Two of the queries, “gut AND microbiome” and “intestine AND microbiome”, retrieved largely overlapping sets of documents, so documents returned by the latter query were not judged. In addition on a second examination of the documents, for the documents judged relevant, she further categorized the documents as relevant for: a) the methodology, b) relation to her specific research question, or c) for her bibliography. Also on this second examination she reviewed documents in greater detail for which she felt less certain of her initial relevance judgments. Some of these original judgments were changed. Of the 401 documents that were judged only 201 were used for the experiments. Some of these documents were duplicates, i.e., retrieved by more than one query, while others were not used because electronic full text versions of the papers were not readily accessible.

To assemble a collection of documents for training and testing the machine learning algorithms, scripts were written which pulled the *html* version of documents available through the *linkout* functionality of PubMed. After stripping the *html* files of markup, stopwords were removed using a standard stopword list. Once stopwords were removed a representation was created for each document. This representation was a vector with a component for each word in the collection. For each component of the vector the corresponding count of the word in that document was entered. This representation was then converted into the ARRF format used by WEKA (see below) to represent feature vectors for machine learning. All experiments were binary categorizations. The documents judged by the researcher to be possibly relevant were alternatively considered relevant, or non-relevant, i.e., we merged the possibly relevant, or “maybe”, documents alternately with either the relevant, or non-relevant, documents as shown in Tables 1 and 2, where these merged documents are shown as “Maybe + yes” and “Maybe + no”.

**Table 1:** Ten-fold cross validation results, *n* = 201

	Maybe + yes – libsvm		Maybes + no – libsvm		Maybe + yes – libsvm - detailed		Maybe + no – libsvm - detailed		Maybe + yes – j48		Maybe + no – j48	
Correctly classified	130	64.68 %	150	74.63 %	97	48.26 %	150	75 %	120	59.70 %	126	62.69 %
Incorrectly classified	71	35.32 %	51	25.37 %	104	51.74 %	50	25 %	81	40.30 %	75	37.31 %
Kappa statistic	0.26		−0.01		−0.04		0		0.14		−0.11	
Mean absolute error	0.35		0.26		0.52		0.25		0.41		0.39	
Root mean squared error	0.59		0.50		0.72		0.5		0.61		0.59	
Relative absolute error	72.11 %		67.64 %		103.48 %		66.42 %		83.59 %		102.51 %	
Root relative squared error	120.09 %		116.52 %		143.86 %		115.47 %		122.76 %		135.82 %

**Table 2:** Training on 2008–2010 documents and testing on 2011 documents, *n* = 19

	Maybe + yes		Maybe + no	
Correctly classified	8	42.11 %	12	63.16 %
Incorrectly classified	11	57.90 %	7	36.84 %
Kappa statistic	0		0	
Mean absolute error	0.58		0.37	
Root mean squared error	0.76		0.61	
Relative absolute error	118.46 %		78.61 %	
Root relative squared error	154.09 %		125.79 %	

Most research on relevance feedback has represented documents using a bag-of-words representation, i.e., wherein a document is represented by the frequencies of words appearing in the document. Using the bag-of-words representation, after common stop words, such as “and” and “the” are removed, a document is represented by the frequencies of the unique words that it contains. These “words” are usually not true words, but rather word stems, such as “comput”, which represents “computer” as well as “computed” or other variations. It is possible to represent a document using a much richer representations using methods from natural language understanding. Such representations might predict relevant documents more accurately, but would also be more expensive computationally to implement. In the future we plan to experiment with richer representations of document content, e.g., based on the concepts contained in the documents as represented in ontologies.

## Results

Table 1 shows results for support vector machines (SVM) (libsvm) and for the C4.5 decision tree algorithm (j48) for the researcher’s initial relevance judgments. All of these results are based on tenfold cross-validation. Both algorithms do well when “maybes” are merged with “noes” and reasonably well, though less so, when “maybes” are merged with “yeses”. Table 1 also shows the results for libsvm when the researcher modified her relevance judgments after making a more detailed examination of the retrieved document, in some cases after reviewing the full text of the document. The columns in Table 1 showing the results based on a more detailed examination of the documents include the word “detailed” in the column heading. Again, results are much better when “maybe’s are combined with “noes”, rather than when combined with “yeses”. Surprisingly, while combining “maybes” with “noes” gives comparable results to the results based on initial relevance judgments, the results given when “maybes” are combined with “yeses” are far worse than was the case with initial relevance judgments.

Table 2 shows the results based on training libsvm on the 2008 – 2010 documents and testing on the 2011 documents. These results are based on the modified, detailed, relevance judgments. While combining “maybes” with “noes” leads to correct classification 63 % of the time, combining “maybes” with “yeses” again gives worse results. These unexpected results are perhaps due to the small size of the test collection, *n* = 19, for the testing on 2011 documents. Our results show the importance of having the researcher express her relevance judgments as “yes”, “maybe”, or “no”. Predicting which documents she will judge relevant, whether based on tenfold cross-validation, or on predicting relevance for 2011 documents based on training on pre-2011 documents, is noticeably more accurate when “maybe” judgments are folded in with “no” judgments than when “maybe” judgments are folded in with “yes” judgments.

## Discussion

### Relevance feedback and improved biomedical information retrieval

In our study we show that relevance feedback from a human microbiome researcher can be used to predict the relevance, for that same researcher, of unseen additional documents. We have not deployed this relevance feedback capability in an information retrieval system, but our task can be seen as a surrogate for the relevance feedback functionality of such a system. In an operational information retrieval system it may not seem practical to ask a searcher to provide relevance judgments for every retrieved document, as we have done in this study. On the other hand, much use has been made of implicit relevance judgments, particularly by Web search engines [40]. Systems using implicit relevance judgments do not ask the searcher which documents are relevant, or not, but rather try to infer relevance from observable user behavior, e.g., how long a user looks at a document or whether the user prints the document. Inferring relevance in this way is a type of clickstream mining [41]. Relevance judgments obtained implicitly are noisier than those obtained by directly asking a user for judgments, but are more practical in practice. Implicit relevance judgments, or clickstream mining, is the technology which underlies auctions of advertising on the Web. Accordingly, it is a well-studied area of research for which effective algorithms have been developed [42]. Our study, using explicit relevance judgments, shows an upper bound on the level of performance that could be expected by noisier implicit feedback. Moreover, in some biomedical information retrieval settings it may be possible to use explicit judgments. Using the approach developed here in a real-world biomedical search system would require training a relevance model for each searcher/information need, as the RefMed [28] system does. Alternatively, implicit relevance feedback might be used to represent the searcher’s information need, as does MiSearch [29]. A next step for future research would be to compare the retrieval effectiveness of explicit relevance feedback for biomedical information retrieval, as explored here, with implicit relevance feedback.

Our results are positive when judgments of maybe relevant are converted to not relevant, i.e., as shown in Tables 1 and 2, where results of 75 % accuracy are achieved using libsvm and tenfold cross validation and 63 %, when training on judgments of 2008–2010 documents to predict relevance for 2011 documents. We believe that better results can be achieved while still using the bag-of-words representation. In our experiments we used the raw frequencies of words in the documents as features. In text retrieval and categorization one usually normalizes word frequencies by document length. Term weights also usually are based on tf*idf, or term frequency/inverse document frequency. The raw, or normalized frequency is the tf component, but idf is important as well. This is the inverse of the proportion of the documents in a collection that contain a term. Thus a term that occurs frequently in a document (tf), but which occurs in a small proportion of the collection (idf) is a good feature.

To obtain substantially better results, though, we believe that richer representations will be needed. The bag-of-words approach can be effective for topic categorization, e.g., that a document is about the microbiome, or is not about the microbiome. For example, in the legal domain one of the authors was able to categorize case law documents as being on the topic, or not, of bankruptcy with a 0.8193 recall rate and a 0.6898 precision rate, though other categories, such as government benefits, were harder with only a 0.2586 recall rate and a 0.3652 precision rate being achieved [43]. Recall is the proportion of all documents of a given category that are categorized as that category by the algorithm, while precision is the proportion of all documents that are categorized as a given category by the algorithm that actually belong to that category. Our task is much harder. We are trying to determine if a document will help meet the information need of a specific researcher, based on positive and negative examples of documents meeting that need. While better feature extraction and weighting at the isolated word level may improve results, we suspect that significantly better results will require an approach based on natural language understanding. We are considering new experiments using the commercial information extraction products, Pathway Studio [44] and Cogito [45]. Pathway Studio is tailored to the biological domain. It extracts entities such as genes and proteins and characterizes the relationship to each other of extracted entities, e.g., up or down regulation. Cogito is a more general purpose package, but it has also been tailored to the biomedical domain. In addition it provides an API, or application programming interface, which allows a user to develop custom information extraction rules.

## Conclusions

Our case study has shown that the relevance feedback judgments of a human microbiome researcher can be used to effectively predict the relevance of additional documents. If these results can be replicated with studies of the relevance feedback judgments provided by additional biomedical researchers, then a proof of concept will be given for the utility of a relevance feedback capability as a component of a biomedical information retrieval system. As mentioned above, such systems are already being used, e.g., RefMed [28].

## Appendix

These were the PubMed queries used for this study.

1. Gut AND microbiome: 305 results
2. Intestine AND microbiome: 257 results (relevance judgments were not made for these documents, as there was significant overlap with the first query)
3. Intestine AND microbiome AND infant: 14 results
4. Intestine AND microbiome AND inflammation: 47 results
5. Intestine AND microbiome AND sepsis: 7 results
6. Intestine AND microbiome AND infection: 28 results

## Abbreviations

API: Application programming interface
ARRF: Attribute-relation file format
EURISCO: European Institute of Cognitive Sciences and Engineering
idf: Inverse document frequency
MEDLARS: Medical literature analysis and retrieval system
NASA: National Aeronautics and Space Administration
TREC: Text REtrieval Conference
SVM: Support Vector Machine
tf: Term frequency
UMLS: Unified Medical Language System
